# Clinical and immunological relevance of SLAMF6 expression in the tumor microenvironment of breast cancer and melanoma

**DOI:** 10.1038/s41598-023-50062-y

**Published:** 2024-01-29

**Authors:** Takaaki Oba, Mark D. Long, Ken-ichi Ito, Fumito Ito

**Affiliations:** 1 Division of Breast and Endocrine Surgery, Department of Surgery, Shinshu University School of Medicine, Matsumoto, Japan; 2 Department of Biostatistics and Bioinformatics, Roswell Park Comprehensive Cancer Center, Buffalo, NY USA; 3 Department of Surgery, Keck School of Medicine, University of Southern California, 1450 Biggy St. NRT 3505, Los Angeles, CA 90033 USA

**Keywords:** Breast cancer, Cancer microenvironment, Tumour immunology

## Abstract

Compelling evidence shows that the frequency of T cells in the tumor microenvironment correlates with prognosis as well as response to immunotherapy. However, considerable heterogeneity exists within tumor-infiltrating T cells, and significance of their genomic and transcriptomic landscape on clinical outcomes remains to be elucidated. Signaling lymphocyte activation molecule 6 (*SLAMF6*) is expressed on intra-tumoral progenitor-exhausted T cells, which exhibit the capacity to proliferate, self-renew and produce terminally-exhausted T cells in pre-clinical models and patients. Here, we investigated the impact of *SLAMF6* expression on prognosis in two immunologically different tumor types using publicly available databases. Our findings demonstrate that high *SLAMF6* expression is associated with better prognosis, expression of *TCF7* (encoding T-cell factor 1), and increased gene signatures associated with conventional type 1 dendritic cells and effector function of T cells in melanoma and breast cancer. Single-cell profiling of breast cancer tumor microenvironment reveals *SLAMF6* expression overlaps CD8 T cells with a T-effector signature, which includes subsets expressing *TCF7*, memory and effector-related genes, analogous to progenitor-exhausted T cells. These findings illustrate the significance of *SLAMF6* in the tumor as a marker for better effector responses, and provide insights into the predictive and prognostic determinants for cancer patients.

## Introduction

Type, density and location of immune cell populations play a critical role in prognosis of various solid malignancies^1^. Particularly, high CD8^+^ T-cell density is one of the most commonly recognized predictive factors of prognosis as well as response to anti-cancer therapy such as chemotherapy^2^ and immune checkpoint inhibitor (ICI) therapy^3^. As ICI therapy becomes the standard-of-care treatment for various cancers; however, we have begun to understand that not only the quantity but also the quality of CD8^+^ tumor-infiltrating lymphocytes (TILs) is critical predictive and prognostic determents. Indeed, whereas PD-1 expression is the hallmark of ‘exhaustion’, a state of dysfunction such as decreased proliferative capacity and effector function^4^, studies have revealed a remarkable degree of heterogeneity with distinct predictive value amongst PD-1^+^ CD8^+^ TILs^5,6^. Of these, two phenotypically and functionally distinct subsets of exhausted T cells have been identified in pre-clinical and human tumors: ‘progenitor-exhausted’ T cells and ‘terminally-exhausted’ T cells. It has been demonstrated that progenitor-exhausted T cells exhibit stem cell-like properties with the capacity to proliferate, self-renew, and produce terminally-exhausted T cells during ICI treatment^7^. Accordingly, the presence of progenitor-exhausted T cells, but not terminally-exhausted T cells, in the tumor microenvironment (TME) is essential to the favorable response to ICIs^7–10^.

Progenitor-exhausted T cells are characterized by the expression of T-cell factor 1 (TCF1, encoded by *TCF7*) and an intermediate level of PD-1 expression, whereas terminally-exhausted T cells do not express TCF1 but harbor high expression of PD-1^10^. TCF1 was found to be expressed by progenitor-exhausted T cells; however, it is also expressed in naïve T cells^11,12^. Hence, it would be difficult to distinguish progenitor-exhausted T cells and other subsets of T cells only with the use of expression level of TCF1 and PD-1.

Signaling lymphocyte activation molecule 6 (SLAMF6, encoded by *Slamf6* in mice, *SLAMF6* in humans) is a homophilic receptor belonging to the superfamily immunoglobulin (Ig) domain-containing molecules, expressed on hematopoietic cells including T, natural killer (NK), and B cells^13,14^ and has emerged as a potential marker for progenitor-exhausted T cells^7,8,15–17^. In preclinical models of melanoma, Slamf6 was identified as a cell-surface marker that distinguished progenitor-exhausted CD8^+^ T cells from terminally exhausted CD8^+^ T cells, and CD8^+^ T cells co-expressing TCF1 and SLAMF6 retained polyfunctionality, produced IFN-γ, TNF, and/or IL-2 and persisted long term upon adoptive transfer, thereby contributing to long-term tumor control compared to terminally-exhausted T cells^8^. Furthermore, our recent work employing single cell (sc) RNA-seq analysis of the TME of murine breast cancer treated with effective multimodal intralesional therapy revealed that *Slamf6* was not expressed on naive or terminally-exhausted T cells^16^, suggesting that *Slamf6* might be a valuable marker to predict response to immunotherapy. However, to date, there has been no study investigating the impact of intra-tumoral SLAMF6 expression on immunological status of the tumor and clinical outcome.

Here, we hypothesize that high expression of *SLAMF6* in the tumor correlates with higher immune activities and better clinical outcome. We investigated the association between expression of *SLAMF6* and immunological status in the tumor and survival in the cohort of breast cancer and melanoma. Using the publicly available data of The Cancer Genome Atlas (TCGA) and single-cell (sc) profiling of breast cancer (GSE161529), our data demonstrate that high expression of *SLAMF6* in the tumor correlates with better patient survival and elevated immune activity in breast cancer and melanoma.

## Results

### High *SLAMF6 *expression is associated with longer progression free interval and overall survival than low *SLAMF6* expression in breast cancer and melanoma

**Figure 1:**
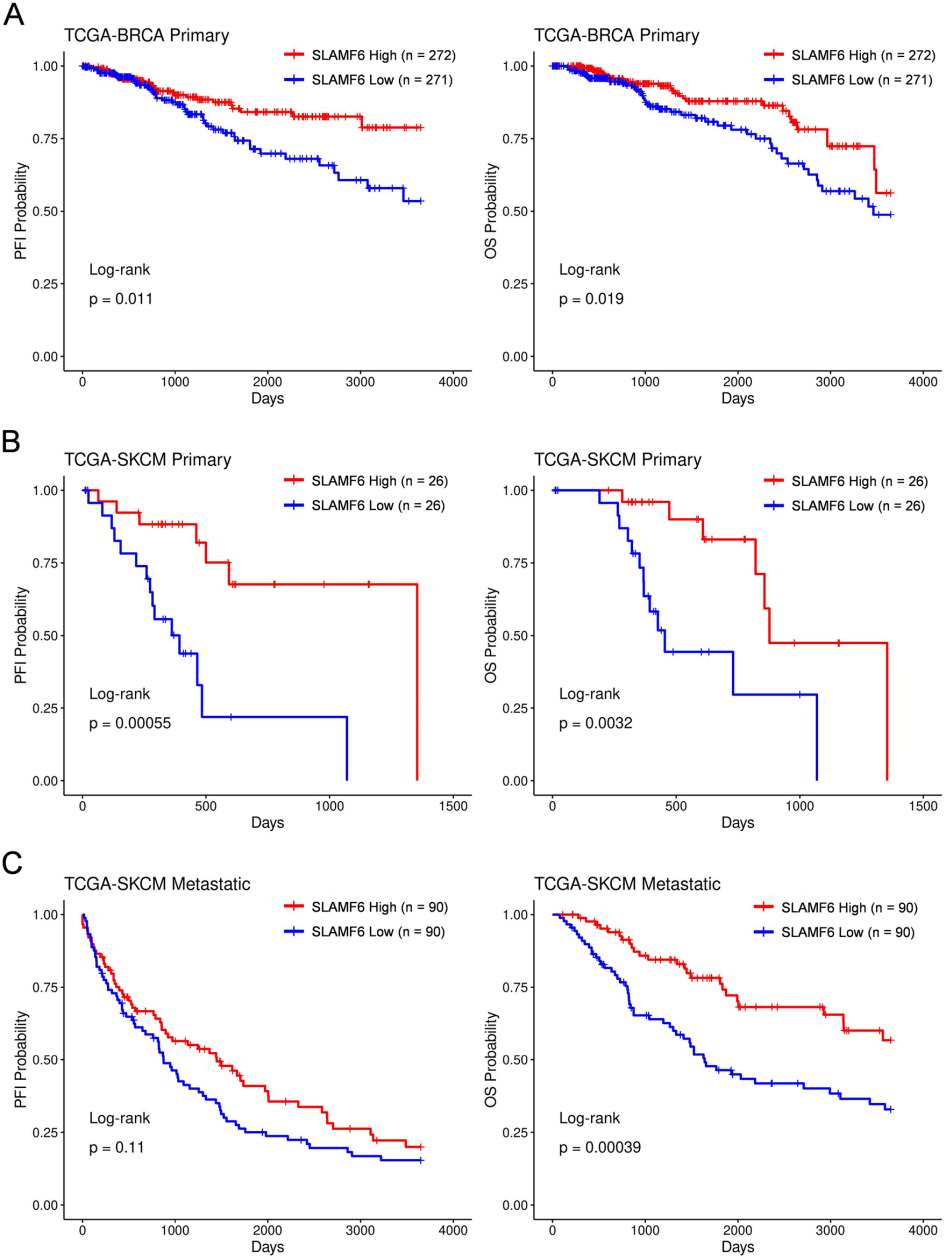
High expression of *SLAMF6* correlates with longer PFI and OS in breast cancer and melanoma. (**A**–**C**) Progression-free interval (PFI) (left) and overall survival (OS) (right) for high versus low *SLAMF6* group in breast cancer (BRCA Primary) (**A**), primary melanoma (SKCM Primary) (**B**) and metastatic melanoma (SKCM Metastatic) cohort (**C**). *P* values were calculated by a log-rank (Mantel–Cox) test (**A**–**C**).

First, we evaluated the prognostic relevance of *SLAMF6* expression on patient outcomes using TCGA database. We analyzed the progression free interval (PFI) and overall survival (OS) of breast cancer and melanoma patients according to the expression level of *SLAMF6*. In the breast cancer cohort, PFI and OS was significantly longer in the high *SLAMF6* group than in the low *SLAMF6* group (PFI; *p* = 0.0011, OS; *p* = 0.0019) (Fig. 1A). In patients with primary melanoma, similarly to the data in breast cancer, the high *SLAMF6* group showed significantly longer PFI and OS than the low *SLAMF6* group in (PFI; *p* = 0.00055, OS; *p* = 0.0032) (Fig. 1B). As for metastatic melanoma, there was a trend that the high *SLAMF6* group had a longer PFI than in the low *SLAMF6* group (*p* = 0.11), while OS was significantly longer in the high *SLAMF6* group than in the low *SLAMF6* group (*p* = 0.00039) (Fig. 1C). Collectively, these findings suggest that higher intra-tumoral *SLAMF6* expression correlates with better prognosis in melanoma and breast cancer patients.

### *SLAMF6* high tumors are enriched with effector T cell- and cDC1-related gene expression in breast cancer and melanoma

**Figure 2:**
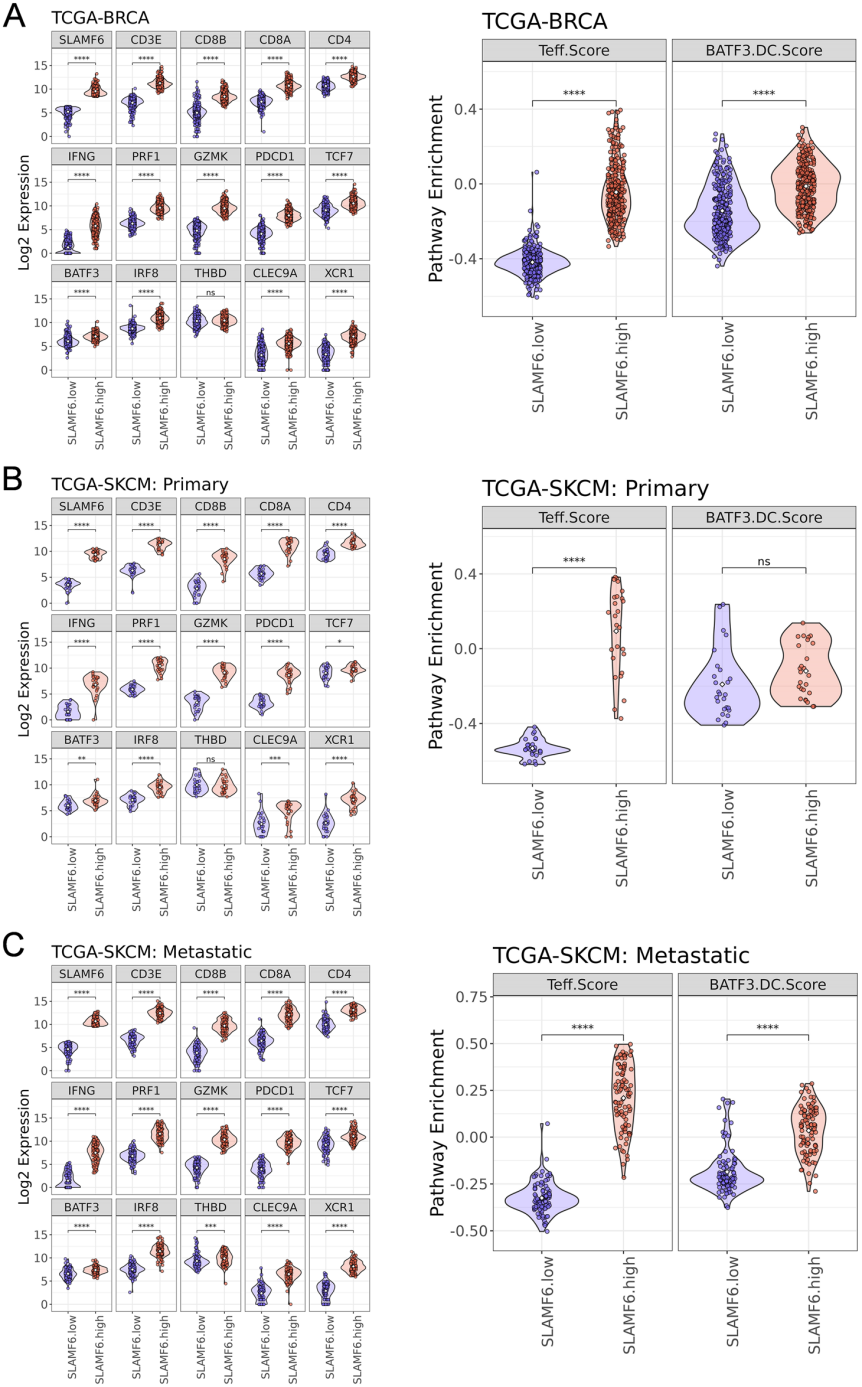
*SLAMF6* high breast cancer and melanoma exhibits high CD8^+^ effector T cell and Batf3-DC Scores. (**A**–**C**) Normalized expression of *SLAMF6, CD3e, CD8b*, *CD8a*, *CD4*, *IFNG*, *PRF1*, *GZMK, PDCD1, TCF7, BATF3, IRF8, THBD, CLEC9A,* and *XCR1* in the *SLAMF6* low and high group (left). Right panels show CD8^+^ effector T cell score (Teff.Score) and Batf3**-**DC score (BATF3-DCScore) in in the *SLAMF6* low and high group. Breast cancer (BRCA) (**A**), primary melanoma (SKCM Primary) (**B**), and metastatic melanoma (SKCM Metastatic) (**C**). *P* values were calculated by a two-tailed Mann–Whitney *U*-test (**A**–**C**).

Next, we investigated the relationship between *SLAMF6* expression and immune activity in the TME of breast cancer and melanoma. We found that *SLAMF6* expression was highly correlated not only with the expression of T-cell markers (*CD3E, CD8B*, *CD8A*, and *CD4*) but also with upregulation of *TCF7, PDCD1* encoding PD-1*, GZMK,* and effector-associated genes including *IFNG* and *PRF1* in breast cancer (Fig. 2A). A previous study has shown that effector T-cell marker gene expression correlates with Batf3-dependent conventional type 1 DC (cDC1) signature in the TME^18^. Therefore, we evaluated expression of *SLAMF6* and cDC1-associated genes (*BATF3*, *IRF8*, *THBD* encoding CD141*, CLEC9A*, and *XCR1*). In breast cancer, cDC1-related genes were substantially upregulated in the high *SLAMF6* group than in the low *SLAMF6* group except for *THBD* (Fig. 2A). Consequently, CD8^+^ T-cell effector (Teff) score, comprising gene expression of *IFNG*, *PRF1, CD8A* and *CD8B,* and BATF3-DC score, calculated with the expression level of *BATF3*, *IRF8*, *THBD, CLEC9A*, and *XCR1*^18^ were markedly increased in the high *SLAMF6* group than in the low *SL AMF6* group in breast cancer. Similar results were observed in the melanoma cohort both in the primary and metastatic settings (Fig. 2B, C).

### *SLAMF6* identifies the immune enriched TME of human breast cancer and melanoma

**Figure 3:**
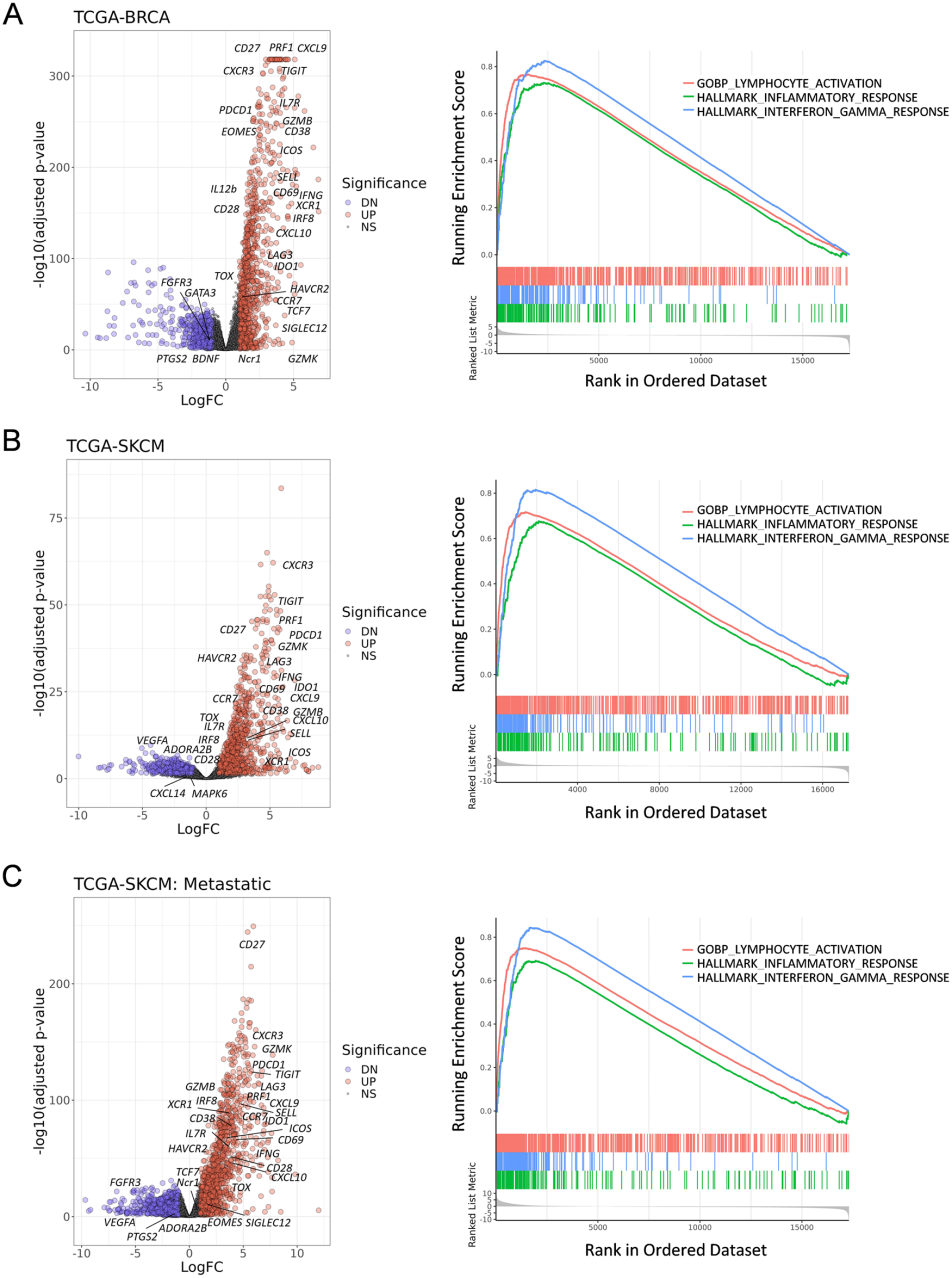
Melanoma and breast cancer with high *SLAMF6* expression are associated with gene expression profiles suggestive of elevated immune activity. (**A**–**C**) Volcano plots (left) showing enrichment differentially expressed genes between the high and low *SLAMF6* group in breast cancer (BRCA) (**A**), primary melanoma (SKCM) (**B**), and metastatic melanoma (SKCM Metastatic) (**C**). Each red and blue dot denotes an individual gene with Benjamini–Hochberg-adjusted *P* value < 0.05 and log fold change > 0.25. Enrichment correlation plots of gene set enrichment analysis (GSEA) between high and low *SLAMF6* expression in the GOBP_LYMPHOCYTE_ACTIVATION (red) HALLMARK_INFLAMMATORY_RESPONSE (green), and HALLMARK_INTERFERON_GAMMA_RESPONSE (blue).

We next examined differentially expressed genes between *SLAMF6* high and low tumors to further elucidate the impact of *SLAMF6* expression on immunological status in the TME. Volcano plots showed that there were a number of differentially expressed genes between *SLAMF6* high and low tumors (Fig. 3A). In addition to the genes presented in Fig. 2A, *SLAMF6* high tumors exhibited high expression of genes and transcripts associated with T-cell activation, effector function, cytotoxicity, exhaustion and memory (*CD27*, *GZMB, CD69, PDCD1*, *TOX, EOMES, LAG3, HAVCR2, IL7R,* and *CD28*) compared with *SLAMF6* low tumors. In contrast, Th2 signature gene, *GATA3* was down regulated in *SLAMF6* high tumors.

We next performed Gene set enrichment analysis (GSEA) of select differential gene sets from three pathway databases (the Hallmark, Canonical pathways, and GO Biological Processes Ontology collections) compiled from the Molecular Signatures Database (MSigDB)^19^. Gene sets associated with inflammatory response, immune response, T-cell activation, and cytokine and IFN signaling pathways were substantially upregulated in breast cancer (Fig. 3A, Supplementary Fig. 1). Similar results were consistently obtained not only in the cohort of primary melanoma but also in that of metastatic melanoma cohort. (Fig. 3B, C, Supplementary Fig. 1).

### Single-cell profiling of the TME of breast cancer identifies SLAMF6^+^ T cells with a genomic signature resembling progenitor-exhausted T cells

**Figure 4:**
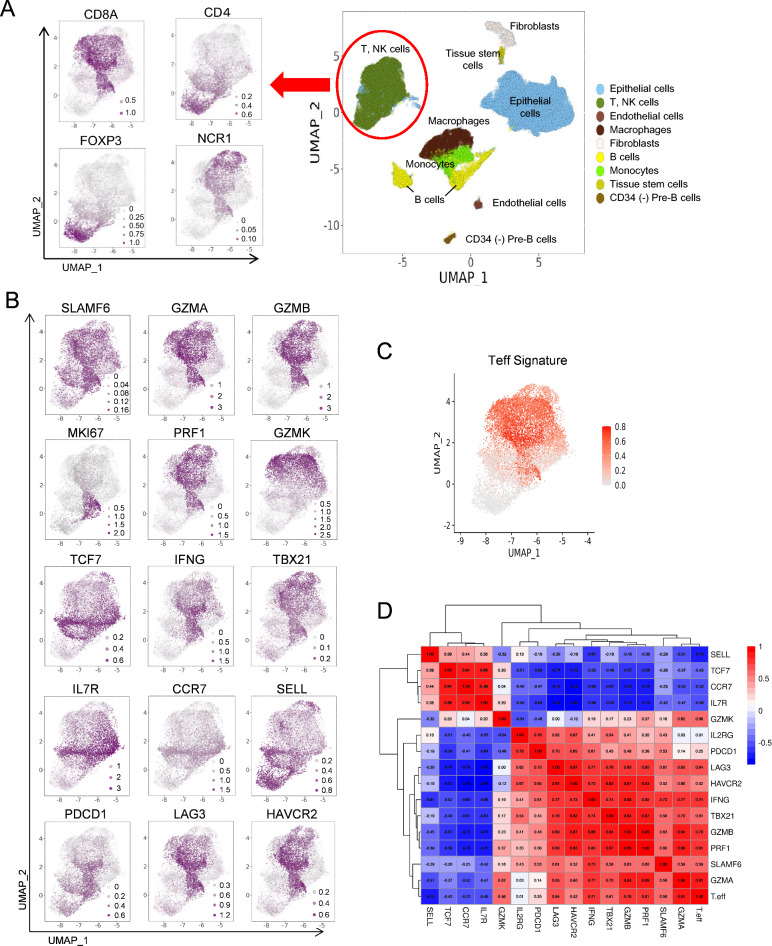
Single cell profile in breast cancer reveals SLAMF6^+^ cells have high capacity of immune response. (**A**) UMAP plots of total cells in breast cancer, and expression plots of indicated genes in T and NK cell population. (**B**) Expression plots of indicated genes in tumor-infiltrating immune cells. Expression levels are color-coded: gray, not expressed; purple, expressed. (**C**) UMAP plots of Teff Signature. Expression levels are color-coded: gray, not expressed; orange, expressed. (**D**) Correlation heatmap showing the Pearson coefficient between SLAMF6 and select gene or Teff signature expressions. Colors range from red for positive correlations, to blue, for negative correlations.

To validate the significance of *SLAMF6* expression in the TME which was obtained from the TCGA database, we next examined *SLAMF6* expression in the scRNA-seq database (GSE161529) of breast cancer^20^. Clustering analysis and subsequent cell type annotation by the human primary cell atlas^21^ revealed 7 clusters (Epithelial cells, T, NK cells, Endothelial cells, Macrophages, Fibroblasts, B cells, and Monocytes) and 2 small clusters of tissue stem cells and CD34(-) Pre-B cells (Fig. 4A). The expression of representative genes (*CD19, CD3E, NCR1, FCGR3A, ITGAM, EPCAM, and ITGA2*) in each cluster are shown in Supplementary Fig. 2A and B. *SLAMF6* was highly expressed in T cells, NK cells, and B cells, but not in epithelial cells, which is concordant with the previous studies^13–15,17,22,23^. This finding indicates that *SLAMF6* is exclusively expressed in immune cells but not in cancerous cells in the TME.

From the initial analysis of total cells, we isolated T and NK cells to evaluate the relation between *SLAMF6* expression and various markers (Fig. 4B), and the T-effector signature defined by the expression level of *CD8A*, *CD8B*, *IFNG*, and *PRF1* (Fig. 4C)^24^. *SLAMF6* expression was predominantly observed in *CD8A*^+^ cells with a high T-effector signature, overlapping with the expression of *GZMA, GZMB, TBX21* encoding T-bet*, PRF1,* and *IFNG*, in agreement with the results from TCGA database (Figs. 2, 3). Yet, *SLAMF6* expression partially overlapped with the expression of *MKI67*, *GZMK*, *TCF7*, memory T cell (*SELL, CCR7,* and *IL7R*)-, and exhaustion-related genes (*PDCD1, LAG3,* and *HAVCR2*).

In line with these findings, while the expression of effector-related genes was markedly associated each other, the correlation between these genes and *SLAMF6* was moderate (Fig. 4D). In addition, the expression of *SLAMF6* was only moderately correlated with exhaustion-related genes. These findings suggest that heterogeneity might exist within *SLAMF6*-expressing T cells in the TME, including subsets co-expressing *SLAMF6*, *TCF7*, memory- and exhaustion-related genes analogous to progenitor-exhausted T cells observed in pre-clinical and human melanomas^7–9^.

## Discussion

In this study, we demonstrate the immunological relevance and prognostic significance of *SLAMF6* expression in the TME of human breast cancer and melanoma. High expression of *SLAMF6* is associated with increased expression of Teff-related genes and *TCF7* which marks tumor-infiltrating T cells with stem cell-like properties^7,8^. Furthermore, high expression of *SLAMF6* is associated with the elevation of anti-cancer immune response represented by the enrichment of gene sets associated with proinflammatory, IFN-γ and T-cell response, and improved survival in patients with breast cancer and melanoma. scRNA-seq analysis of existing human breast cancer dataset demonstrates that subsets of T cells co-expressing *SLAMF6*, *TCF7*, memory- and exhaustion-related genes comparable to progenitor-exhausted T cells identified in pre-clinical models and human melanomas^7–9^. Melanoma is known to harbor one of the highest somatic mutation frequencies among human solid malignancies^25^. Subsets of somatic mutations can create tumor-specific antigens which are recognizable by the immune system. Hence, melanoma has been considered immunogenic tumors, and the targets of immunotherapy. High mutational burden of melanoma is associated with enriched T-cell infiltration, and such T-cell inflamed tumors are referred as immunologically ‘hot’ tumors^26^. In contrast, breast cancer is characterized with infrequent somatic mutation or poor T-cell infiltration^25,27^, and is often called as immunologically ‘cold’ tumors. The results of this study showed that *SLAMF6* expression was associated with elevated anti-cancer immune activity and better prognosis both in the ‘cold’ tumor (breast cancer) and the ‘hot’ tumor (melanoma). Although we could not investigate the association between *SLAMF6* expression and response to ICIs, *SLAMF6* expression might be a promising biomarker that can be useful in predicting response to ICIs. Further studies to address this point are warranted.

A large body of evidence has shown the critical roles of the cross-presenting DCs to elicit tumor-specific T-cell immunity, increase TILs and improve the treatment outcome of ICIs in mouse melanoma models and patients with this disease^18,28–30^. A previous study also showed the key role of tumor-residing cDC1s in facilitating trafficking of effector T cells into the TME via chemokine/chemokine receptor axis^18^. Furthermore, a recent study demonstrated that cDC1s could traffic to tumor-draining lymph nodes, and become a continual source of generating Tcf1^+^ CD8^+^ T cells^31^. In accordance with this scenario, our studies recently demonstrated that induction and activation of tumor-residing cDC1s could promote generation of tumor-specific T cells, convert poorly T cell-infiltrated tumors into infiltrated TMEs, facilitate an influx of Slamf6^+^ Tcf1^+^ T cells into tumors, induce not only the regression of primary but also untreated distant lesions, and overcome resistance to ICIs using mouse models of melanoma and breast cancer^16,32–34^.

In the present study, in order to examine whether *SLAMF6* expression might correlate with the presence of cDC1s in the tumor, we evaluated the expression of *SLAMF6* and BATF3 scores^18^. Our findings showed that expression of cDC1-related genes were significantly increased in the high *SLAMF6* group than in the low *SLAMF6* group, indicating that *SLAMF6* expression in the tumor correlates with cDC1s density in the tumor. These findings are concordant with the results from the previous studies that tumor-residing cDC1 plays a critical role in trafficking as well as generation of tumor-specific T cells to the TME^16,18,29,35^. Given that expression of *TCF7* and Teff score were significantly elevated in the high *SLAMF6* group than in the low *SLAMF6* group, it is conceivable that there might be a correlation of cDC1 and progenitor-exhausted T cells expressing *SLAMF6*. Of note, these prior results were obtained from preclinical model or melanoma patients^18,29,31^. Hence, this study provided the novel insight into the correlation between the cDC1 signature and markers of progenitor-exhausted T cells even in the immunologically ‘cold’ tumors.

Although ICI therapy has become a standard treatment in the management of a variety of solid malignancies including melanoma and breast cancer, many patients do not respond. PD-L1 expression in the TME correlates with increased response to PD-1/PD-L1 blockade therapy^36^; however, PD-L1 expression status alone does not appear to be a useful biomarker to select patients for the treatment because PD-L1 negative tumors often show dramatic response. Discovery of additional pre-treatment tumor biomarkers may lead to better identify patients who could get benefit from ICI therapy. TCF1 and SLAMF6 are both recognized as markers for progenitor-exhausted T cells^8,10^, and hence it would be expected that the expression of *TCF7* is positively correlated with that of *SLAMF6*. Indeed, analysis of TCGA data demonstrated that *TCF7* expression was higher in the *SLAMF6* high tumors than in the *SLAMF6* low tumors both in breast cancer and melanoma. This was consistent with the finding from our scRNA-seq analysis which identified *SLAMF6*^+^ cells expressing *TCF7*, effector- and memory-related genes, compatible to the subset of progenitor-exhausted T cells identified in the TME of melanoma patients^7–9^. We further found that heterogeneity within the *SLAMF6*^+^ subsets might exist, which include a population expressing high levels of *MKI67* resembling *Slamf6*^+^ *Ki67*^+^ cells displaying vigorous expansion in response to viral infection in pre-clinical models^37,38^. Collectively, these findings align with previous studies showing that *SLAMF6*^+^cells are poised for better effector responses^7,8^.

Although SLAMF6 was found to be expressed in mouse and human progenitor-exhausted T cells in the TME, and associated with enhanced effector responses^7–9,15–17^, SLAMF6 may hold an inhibitory function. Previous studies have shown that anti-SLAMF6 antibody reduced the tumor burden in preclinical models of leukemia and melanoma through the activation of CD8 T cells^17^, and knock-out of *Slamf6* in anti-melanoma CD8^+^ T cells improved therapeutic efficacy of adoptive T cell therapy^23^. Furthermore, Eisenberg et al. reported that the use of the soluble ectodomain of SLAMF6 which enhances receptor dephosphorylation reduced activation-induced cell death, increased IFN-γ production and cytotoxicity in tumor-specific CD8 T cells, resulting in improved tumor control in preclinical models of melanoma^39^. Therefore, accumulating evidence indicates that SLAMF6 may act as T-cell inhibitory receptor, and is not only expressed in progenitor-exhausted T cells but also various states of T cells, which is in line with our findings from scRNA-seq analysis. In our study, we found the association of SLAMF6 expression with augmented effector signature and improved prognosis. However, the exact mechanisms of how SLAMF6^+^ T cells contribute to these effects remain to be elucidated. Indeed, it's essential to understand the precise intracellular signaling pathways induced by SLAMF6 engagement in the tumor microenvironment, especially if therapeutic targeting is considered. To this end, further mechanistic studies that can elucidate the signaling dynamics of SLAMF6 are warranted.

This study had several limitations. First, this is a retrospective study using a previously collected cohort of patients, and selection bias in patient background should be noted. TCGA database contains patients who underwent various treatments. Hence, it should be noted that the link between *SLAMF6* expression and improved survival is not associated with specific therapy. Furthermore, it is unclear whether the patients in our cohorts were treated with ICIs. Thus, it remains unknown whether *SLAMF6* expression is associated with response to ICI therapy. Second, the analyses in our study were based on mRNA expression data, which is not fully concordant with protein expression. Further study is warranted to investigate the protein expression of SLAMF6 using immunohistochemistry. Lastly, the sample size in GSE161529 is relatively small, which potentially limits the generalizability of our findings. Furthermore, pooled analysis using 8 patients signals would not properly reflect the individual-level association. Further validation including individual-based analysis in a larger cohort would strengthen our findings.

In summary, the results of this study demonstrate that high expression of *SLAMF6* in TME correlates with elevated immune activities and better prognosis both in breast cancer and melanoma. Further study may reveal that *SLAMF6* expression can be a biomarker predicting response to ICIs.

## Methods

### Analysis of TCGA melanoma and breast cancer cohorts

TCGA-BRCA and TCGA-SKCM expression and clinical annotations were obtained from the Genomic Data Commons data portal and processed via TCGAbiolinks package in R using TCGAWorkflow guided practices^40^. Differential expression associated with SLAMF6 expression (SLAMF6-high = top quartile, SLAMF6-low = bottom quartile) within each respective cohort was determined by TCGAbiolinks/edgeR. Gene set enrichment analysis (GSEA) of ranked differential expression was assessed using the clusterProfiler package against gene sets derived from the Hallmark, Canonical pathways, and GO Biological Processes Ontology collections retrieved from the MSigDB^41^. Enrichment of gene sets reflecting the presence of CD8^+^ effector T-cells (*CD8A*, *CD8B*, *IFNG*, *PRF1*) and BATF3 DCs (*BATF3*, *IRF8*, *THBD*, *CLEC9A*, *XCR1*) were determined by ssGSEA using the GSVA package^42^. Progression free interval (PFI) and overall survival (OS) between SLAMF6-high and SLAMF6-low tumors was performed using the survival package.

### Analysis of TNBC single-cell RNA-sequencing data

Raw scRNA-seq counts derived from cells captured from 8 TNBC samples^20^ were obtained directly from the gene expression omnibus (GSE161529). Filtering, normalization, and downstream analyses including variable feature selection, dimensionality reduction (PCA), uniform manifold approximation and projection (UMAP) low-dimensional representation, and kNN based clustering were performed using Seurat (v3)^43^. Cells were annotated to major cell lineages defined in the human primary cell atlas using singleR^21^. Cell gene expression was imputed using MAGIC^44^ implemented via the Rmagic package. T-cells were filtered from total cells for assessment of SLAMF6 in relation to various markers of T-cell phenotype. Single-cell pathway enrichment for CD8^+^ effector T-cells (*CD8A*, *CD8B*, *IFNG*, *PRF1*) was performed by UCell, and Teff Signature was calculated^24^. Associations between SLAMF6 expression and select genes or signatures within T-cells were examined by Pearson correlation analysis.

### Supplementary Information

Supplementary Legends.

Supplementary Figure 1.

Supplementary Figure 2.

## Data Availability

The datasets analyzed during the current study are available at https://portal.gdc.cancer.gov/projects/TCGA-BRCA (TCGA-BRCA) and https://portal.gdc.cancer.gov/projects/TCGA-SKCM (TCGA-SKCM). Raw scRNA-seq counts were obtained directly from the GEO under the accession number GSE161529 (https://www.ncbi.nlm.nih.gov/geo/query/acc.cgi?acc=GSE161529). The data that support the findings of this study are publicly available from the corresponding author upon reasonable request and can be down.
