# Supplementary material for: Clinical and immunological relevance of SLAMF6 expression in the tumor microenvironment of breast cancer and melanoma
Source: Sci Rep. 2024 Jan 29;14:2394. doi: 10.1038/s41598-023-50062-y (PMC10825192; doi:10.1038/s41598-023-50062-y)
Supplement: Supplementary file 1 — Supplementary Legends. [file 41598_2023_50062_MOESM1_ESM.docx]

**Supplementary figure legends**

**Supplementary Fig. 1 GSEA shows that melanoma and breast cancer with high *SLAMF6* expression have elevated expression associated with inflammatory, T-cell and immune response and/or cytokine and IFN signaling pathways.**

Emap plots (left) showing normalized enrichment score of each pathway. Each dot denotes a pathway with Benjamini–Hochberg-adjusted P- value <0.05 and log fold change >0.25. Red dot indicates upregulation in SLAMF6 high tumors, while blue indicates downregulation. Normalized enrichment score in selected pathways from the Hallmark, Canonical pathways, and GO Biological Processes Ontology collections in the high and low *SLAMF6* group.

**Supplementary Fig. 2 Gene expression according to the cell type**

**A** Expression of *CD19*, *CD3E*, *NCR1*, *FCGR3A*, *ITGAM*, *EPCAM*, *ITGA2*, and *SLAMF6* in each cell type as indicated. **B** Heatmap displaying normalized expression of select genes in each cell type.
