# Supplementary figures and images for: Clinical and immunological relevance of SLAMF6 expression in the tumor microenvironment of breast cancer and melanoma
Source: Sci Rep. 2024 Jan 29;14:2394. doi: 10.1038/s41598-023-50062-y (PMC10825192; doi:10.1038/s41598-023-50062-y)

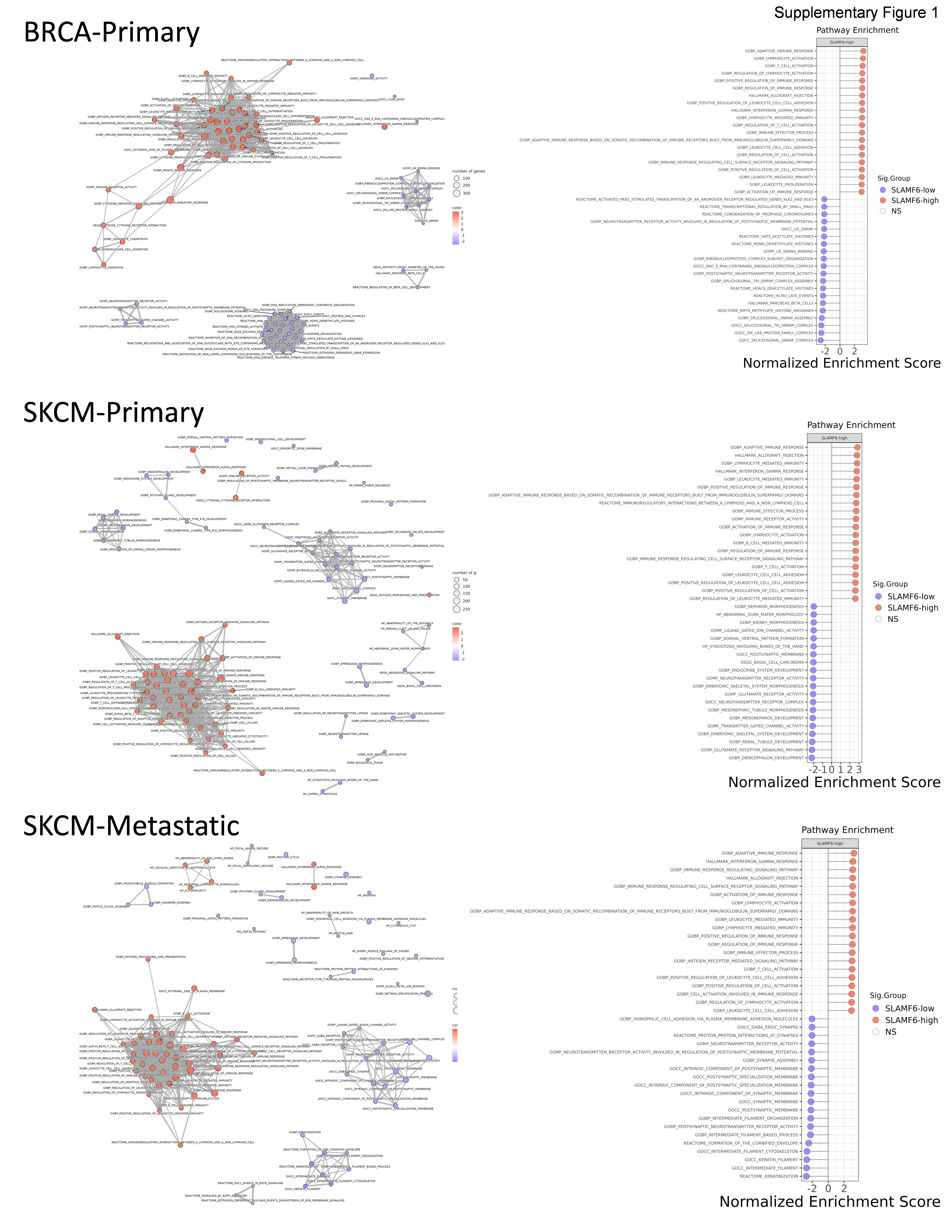

Supplement: Supplementary file 2 — Supplementary Figure 1. [file 41598_2023_50062_MOESM2_ESM.tif]

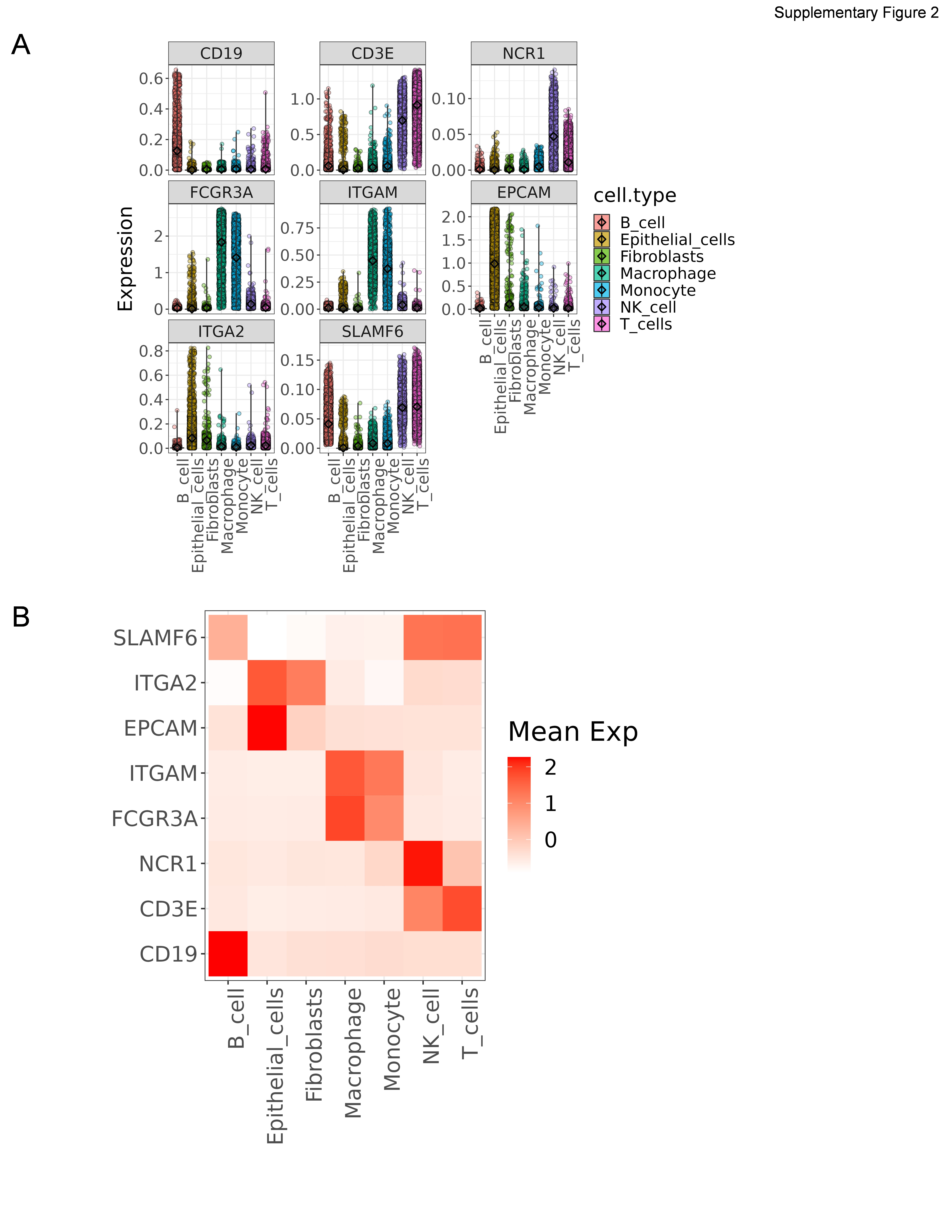

Supplement: Supplementary file 3 — Supplementary Figure 2. [file 41598_2023_50062_MOESM3_ESM.tif]
